# Polytetrafluorethylene-Au as a substrate for surface-enhanced Raman spectroscopy

**DOI:** 10.1186/1556-276X-6-366

**Published:** 2011-04-28

**Authors:** Pavel Žvátora, Pavel Řezanka, Vadym Prokopec, Jakub Siegel, Václav Švorčík, Vladimír Král

**Affiliations:** 1 Department of Analytical Chemistry, Institute of Chemical Technology Prague, Technická 5, 16628, Prague 6, Czech Republic; 2 Department of Solid State Engineering, Institute of Chemical Technology Prague, Technická 5, 16628 Prague 6, Czech Republic

## Abstract

This study deals with preparation of substrates suitable for surface-enhanced Raman spectroscopy (SERS) applications by sputtering deposition of gold layer on the polytetrafluorethylene (PTFE) foil. Time of sputtering was investigated with respect to the surface properties. The ability of PTFE-Au substrates to enhance Raman signals was investigated by immobilization of biphenyl-4,4'-dithiol (BFD) from the solutions with various concentrations. BFD was also used for preparation of sandwich structures with Au or Ag nanoparticles by two different procedures. Results showed that PTFE can be used for fabrication of SERS active substrate with easy handle properties at low cost. This substrate was sufficient for the measurement of SERS spectrum of BFD even at 10^-8 ^mol/l concentration.

## Introduction

Surface-enhanced Raman scattering (SERS) has great potential as an analytical technique based on the surface enhancement of Raman signals of the molecule situated on the metal surface which is nowadays currently used for the detection of various analytes at low concentration [1]. In general, there are two traditional operational mechanisms to describe the overall SERS effect: electromagnetic [2] and chemical [1,2] enhancement mechanism. Electromagnetic mechanism lies in the enhancement of local electromagnetic field of incident radiation applied on the molecule which is adsorbed on or situated in the close proximity to rough metal surface. In order to obtain optimal enhancement of Raman signals of the molecule it is necessary to use nanostructured surfaces or nanoparticles of noble metals with suitable physical parameters such as their size, shape, and degree of aggregation [3]. Many different types of SERS substrates, which meet the above requirements have been developed, including roughened electrodes [4,5], noble metal colloidal nanoparticles [6,7], silver island films [8,9], metal film over nanostructured surfaces [10,11], acid-etched metal foils [12], and lithographically produced nanoparticle arrays [13,14]. Plastic substrates are also known [15]. Polymers were commonly used for improved mechanical stability of nanoparticles [16] and better signal reproducibility via embossing surfaces and lithographic techniques [15,17]. Polytetrafluorethylene (PTFE) is a polymer with broad potential applications in microelectronics. Another advantage of this material is its high thermal stability and low degradation due to the exposition to a focused laser beam. PTFE foil has great surface roughness with improved adhesive properties of sputtering gold over layer and can be positive for electromagnetic mechanism. Gold over layer can suppress Raman background signal of the PTFE substrate [15].

Within the experiments described in this study we have prepared suitable SERS active substrates from synthetic polymer foils of PTFE by deposition of Au layers on its surface inside of plasma discharge [18]. Electromagnetic mechanism enhancement was tested on rude PTFE-Au surface and sandwich structures. The fabrication of sandwich structures [19] was realized by incorporating of self-assembled monolayer of dithiols between the layers of PTFE-Au surface and Au or Ag nanoparticles.

## Experimental

### Preparation of gold layer on PTFE foil

**Figure 1:**
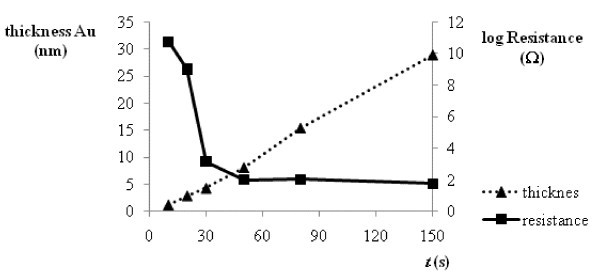
**Dependence of the thickness of gold layer on time of sputtering (dash line) and resistance values of this gold layer (solid line)**.

The gold layers were sputtered on PTFE foils (2 cm in diameter) with a thickness of 50 μm. The time of sputtering was from 10 to 150 s and deposition parameters were described elsewhere [20]. Microbalance was used for gravimetric determination of the amount of sputtered gold on polymeric substrate. Continuity of sputtered gold layer was determined by measuring of its resistance by the picoapermeter (Figure 1).

The influence of time of sputtering (*t *= 10, 20, 30, 50, 80, 150 s) and concentration (*c *= 10^-2^, 10^-3^, 10^-4^, 10^-5^, 10^-6^, 10^-7^, 10^-8 ^mol/l) of used bifunctional compound (biphenyl-4,4'-dithiol) on the intensity of SERS signals was then studied. In order to study the sputtering time gold layers were modified with biphenyl-4,4'-dithiol in methanol solutions (10^-2 ^mol/l). PTFE foil with gold layer was placed into the methanol solution for 12 h. After that the foil was taken out from the solution, washed by pure methanol, and dried on the air. The study of concentration dependence was similar.

### Preparation of nanoparticles

Gold nanoparticles (AuNPs) were obtained by citrate reduction of K[AuCl_4_] described elsewhere [21]. Silver nanoparticles (AgNPs) were obtained using similar process of AgNO_3 _reduction published by Smitha et al. [22]. Prepared nanoparticles were characterized by TEM and UV-Vis absorption spectroscopy. UV-Vis absorption spectroscopy was carried out using a Varian spectrophotometer, model Cary 400 SCAN, from 200 to 800 nm. The transmission electron microscopy (TEM) images were recorded using a JEOL microscope, model JEM-1010 with accelerating voltage 100 kV.

### Preparation of sandwich structures

**Figure 2:**
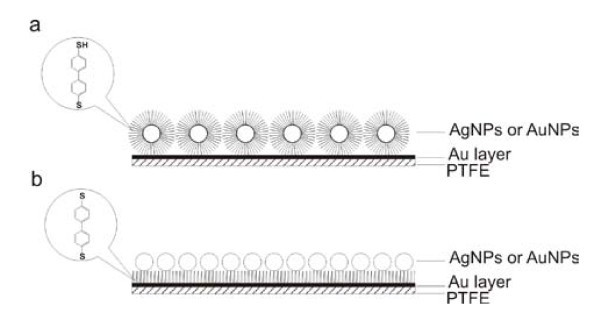
**(a) Preparation of sandwich structures using modified nanoparticles**. **(b) **Preparation of sandwich structures using modified gold layer.

The sandwich structures were prepared by two procedures. In the first one (Figure 2a), the gold foils were modified by silver or gold nanoparticles previously covered by biphenyl-4,4'-dithiol. 1 ml of nanoparticles was added drop-wise at intensive stirring to the 1 ml of biphenyl-4,4'-dithiol solution with concentration of 5 × 10^-2 ^mol/l. The obtained mixture was purified by centrifugation. PTFE foil with gold layer was placed to the solution of nanoparticles for 12 h. After that the foil was removed from the solution, washed by pure methanol, and dried on the air.

In the second procedure (Figure 2b), PTFE foil with gold layer modified by biphenyl-4,4'-dithiol was prepared. Then such modified foil was placed into the solution of 2 ml of nanoparticles for 12 h. After that the foil was removed from the solution, washed by pure methanol, and dried on the air.

### SERS measurements

Raman spectral measurements were performed on Raman NIR Advantage spectrograph DeltaNu with laser excitation line 785 nm, power 100 mW in the range of 100 to 2000 cm^-1 ^with spectral resolution 4 cm^-1^. Integration time was 20 s and results spectra are average of five measurements. Surface was focused by the NuScope with manual adjustment and field of view was approximately 800 μm at 100 × focal power. All measurements were carried out on two different places from both sides of PTFE foil.

## Results and discussion

### Properties of prepared gold layers on PTFE foil

The results of measurements of prepared gold layers on PTFE foils are shown in Figure 1. The thickness of gold layer was calculated from the mass difference of foils before and after sputtering procedure. It is clear from the table that the thickness is a linear function of sputtering time. The value of resistance is related to continuity of gold layer [18]; therefore, when short times are applied the resistance values are very high and the layer is discontinual, while after the applications of longer times the resistance values change to low which means that the layer becomes continual.

### Preparation of nanoparticles

**Figure 3:**
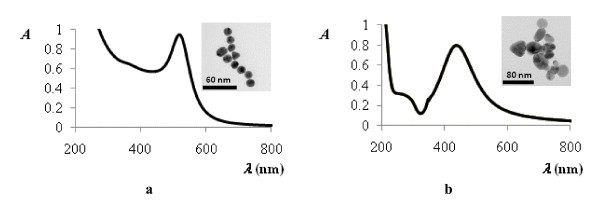
**(a) UV-Vis absorption spectra of the AuNPs and TEM image (in excision); (b) UV-Vis absorption spectra of the AgNPs and TEM image (in excision)**.

The average diameter of the prepared spherically shaped AuNPs electrostatically stabilized with citrate was about 15 nm and for AgNPs was about 45 nm. The wavelengths of their surface plasmon absorbance maximums (AuNPs at 520 nm and AgNPs at 430 nm) correspond well with the averages diameters estimated by TEM [21,23] (Figure 3).

### SERS measurements on PTFE foils

**Figure 4:**
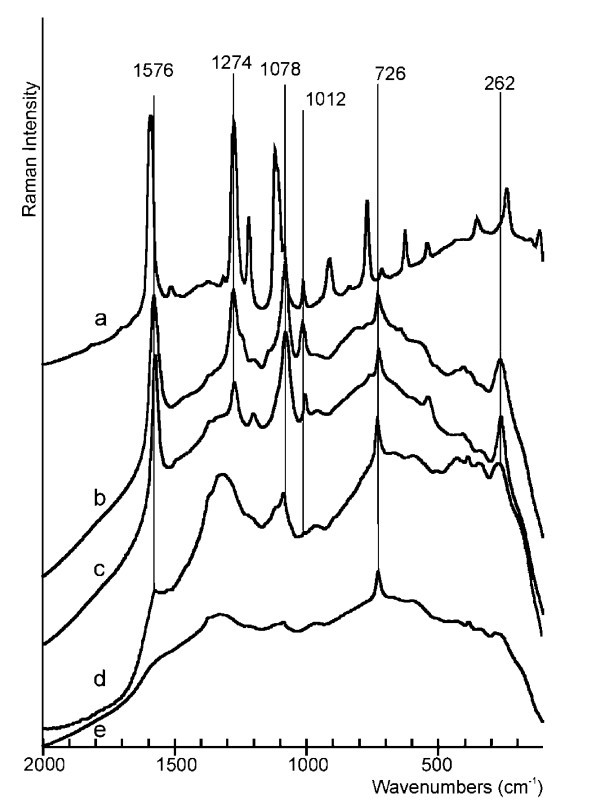
**(a) Raman spectrum of pure BFD; (b) SERS spectrum of immobilized BFD (*c *= 10^-2 ^mol/l) on the PTFE-Au; (c) SERS spectrum of immobilized BFD (*c *= 10^-8 ^mol/l) on the PTFE-Au with AgNP (prepared by Figure 2b); (d) SERS spectrum of immobilized BFD (*c *= 10^-8 ^mol/l) on the PTFE-Au with AuNP (prepared by Figure 2b); (e) Raman spectrum of pure PTFE**.

We have chosen biphenyl-4,4'-dithiol (BFD; Figure 4a) as compound for the immobilization on the PTFE foil with gold layer (PTFE-Au) due to possibility of linking it into sandwich structures. In contrast to other commercially available dithiols (i.e., ethan-1,2-dithiol, hexan-1,6-dithiol), BFD has a rigid structure that provides such orientation on the surface that the possibility of binding by both of sulfonyl groups is very improbable.

**Figure 5:**
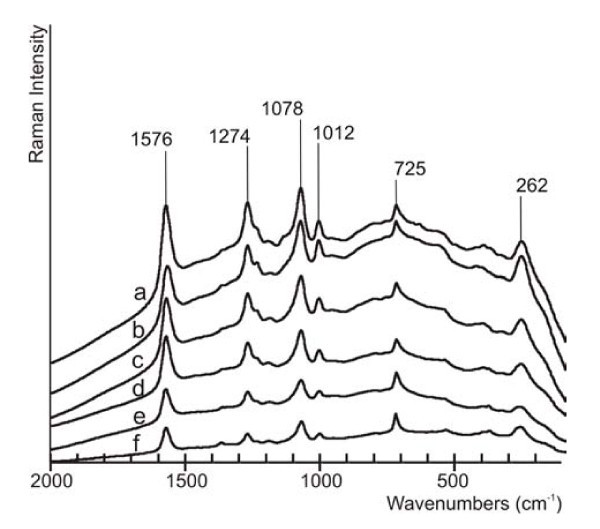
**SERS spectra of BFD on PTFE-Au for (a) 30 s, (b) 20 s, (c) 50 s, (d) 80 s, (e) 150 s, (f) 10 s sputtering time**. Spectra were shifted in *y*-axis.

For the evaluation of the dependence of sputtering time on the quality of SERS spectra we choose the band at 1078 cm^-1 ^due to its high intensity and the possibility of easy baseline correction. The dependence of area of this signal on the sputtering time (Figure 5) showed that the maximal intensity of SERS signal was achieved using 30 s of the sputtering time (Figure 4b). According to Figure 1, at this time the layer is changing from discontinuous to continuous (see resistance). Due to this fact, when time of sputtering shorter than this is applied, the surface of gold layer is so much discontinuous that surface enhancement of Raman signals is very small and, on the contrary, after the application of longer sputtering time, the surface of gold layer is too much continuous, which leads to small enhancement because the surface has not got an optimal roughness. The analytical enhancement factor was calculated from the ratio of band intensity (1078 cm^-1^) of pure BFD solution (*c *= 1 × 10^-2 ^mol/l) in CHCl_3 _and BFD (*c *= 1 × 10^-8 ^mol/l) immobilized on PTFE-Au without and with nanoparticles (Table 1).

**Table 1:** The analytical enhancement factor of the surface for immobilized BFD (calculated for *c *= 1 × 10^-^^8 ^mol/l); sandwich structures were prepared according to Figure 2b

Type of surface	Analytical enhancement factor
PTFE-Au-BFD	3.89 × 10^5^

PTFE-Au-BFD-AuNP	9.12 × 10^5^

PTFE-Au-BFD-AgNP	6.73 × 10^6^

In the second step, we investigated the effect of concentration of BFD solution, the type of metal nanoparticles and the effect of their immobilization on the intensities of SERS signals of PTFE-Au prepared by sputtering with the duration of 30 s. The results (Table 2) show that the maximum intensity of the selected band was achieved with BFD concentration of 10^-6 ^mol/l. The effect of sandwich structure prepared according to procedures which is showed in Figure 2b caused the enhancement of the signal even at lower concentrations, so we obtained SERS spectrum of AgNPs covered by BFD even at 10^-8 ^mol/l (Tables 1, 2; Figure 4c). The signal at 726 cm^-1 ^(spectrum 4b, 4c, 4d, and 4e) corresponds to deformation vibration of CF_2 _group of PTFE. The preparation of sandwich structures by the other procedure (Figure 2a) led to obtainment of similar spectra and the influence of the type of metal nanoparticles was negligible based on the identical SERS spectral structure. We propose that it is due to the fact that the concentration of BFD immobilized on the nanoparticles is similar.

**Table 2:** The dependence of the area of the selected peak 1078 cm^-^^1 ^in SERS spectra on the concentration of BFD

*c *(BFD)/mol/l	PTFE-Au-BFD	PTFE-Au-BFD-AuNP (prepared according to Figure 2b)	PTFE-Au-BFD-AgNP (prepared according to Figure 2b)
10^-2^	3.21 × 10^6^	1.44 × 10^6^	1.86 × 10^6^

10^-3^	8.37 × 10^6^	4.09 × 10^6^	6.10 × 10^6^

10^-4^	5.96 × 10^6^	4.12 × 10^6^	5.93 × 10^6^

10^-5^	10.3 × 10^6^	8.91 × 10^6^	8.04 × 10^6^

10^-6^	12.6 × 10^6^	9.06 × 10^6^	4.96 × 10^6^

10^-7^	6.38 × 10^6^	0.709 × 10^6^	6.58 × 10^6^

10^-8^	0.09 × 10^6^	0.218 × 10^6^	1.14 × 10^6^

It was found that the enhancement of Raman signals of BFD is independent on the measured side of PTFE foil due to the transparency of the foil and very thin layer of sputtered gold. Further, reproducibility of foil preparation is very high but the reproducibility of BFD- and NPs-modified foils is lower (RSD = 20%).

## Conclusions

In summary, we have demonstrated the possible preparation of SERS active substrate with suitable properties by sputtering deposition of gold layer on the PTFE foil. Such foil is cheap, easy to manipulate with it, and offers the possibility to measure from both sides of PTFE foil. It was found out that optimum of sputtering time is for 30 s and the maximum of SERS signal intensity was achieved at 10^-6 ^mol/l for BFD. With use of sandwich structures of nanoparticles we were able to obtain signal even at 10^-8 ^mol/l. This substrate had the highest analytical enhancement factor (6.73 × 10^6^).

## Abbreviations

BFD: biphenyl-4,4'-dithiol; PTFE: polytetrafluorethylene; SERS: surface enhanced Raman spectroscopy; TEM: transmission electron microscopy.

## Competing interests

The authors declare that they have no competing interests.

## Authors' contributions

PŽ was responsible for synthesis and other characterizations of nanomaterials (AuNPs, AgNPs and sandwich structures), writing up of this manuscript and participated in interpretation of experimental data. PŘ and VP were responsible for recording SERS spectra and interpretation of this data. JS and VŠ carried out part of the preparation and characterization of Polytetrafluorethylene-Au substrates. VK is the supervision and participated in the results, discussion and manuscript revision. All authors read and approved the final manuscript.
